# Brain structure, cognition and negative symptoms in schizophrenia are associated with serum levels of polysialic acid-modified NCAM

**DOI:** 10.1038/tp.2015.156

**Published:** 2015-10-13

**Authors:** F Piras, M Schiff, C Chiapponi, P Bossù, M Mühlenhoff, C Caltagirone, R Gerardy-Schahn, H Hildebrandt, G Spalletta

**Affiliations:** 1 Department of Clinical and Behavioral Neurology, Neuropsychiatry Laboratory, IRCCS Santa Lucia Foundation, Rome, Italy; 2 Institute for Cellular Chemistry, Hannover Medical School, Hannover, Germany; 3 Department of System Medicine, Tor Vergata University, Rome, Italy; 4 Division of Neuropsychiatry, Menninger Department of Psychiatry and Behavioral Sciences, Baylor College of Medicine, Houston, TX, USA

## Abstract

The neural cell adhesion molecule (NCAM) is a glycoprotein implicated in cell–cell adhesion, neurite outgrowth and synaptic plasticity. Polysialic acid (polySia) is mainly attached to NCAM (polySia-NCAM) and has an essential role in regulating NCAM-dependent developmental processes that require plasticity, that is, cell migration, axon guidance and synapse formation. Post-mortem and genetic evidence suggests that dysregulation of polySia-NCAM is involved in schizophrenia (SZ). We enrolled 45 patients diagnosed with SZ and 45 healthy individuals who were submitted to polySia-NCAM peripheral quantification, cognitive and psychopathological assessment and structural neuroimaging (brain volumes and diffusion tensor imaging). PolySia-NCAM serum levels were increased in SZ patients, independently of antipsychotic treatment, and were associated with negative symptoms, blunted affect and declarative memory impairment. The increased polySia-NCAM levels were associated with decreased volume in the left prefrontal cortex, namely Brodmann area 46, in patients and increased volume in the same brain area of healthy individuals. As this brain region is involved in the pathophysiology of SZ and its associated phenomenology, the data indicate that polySia-NCAM deserves further scrutiny because of its possible role in early neurodevelopmental mechanisms of the disorder.

## Introduction

Development of brain connectivity requires temporal and spatial control of cellular interactions. The neural cell adhesion molecule (NCAM) is a member of the immunoglobulin superfamily and is a key factor in these processes, including axonal/dendritic growth and branching and synaptic plasticity.^1^ In vertebrates, NCAM can be modified by polysialic acid (polySia), a linear homopolymer of sialic acids that is mainly present during fetal and early postnatal brain development.^2, 3^ PolySia disappears almost completely from the adult brain, except for some areas of continuous neurogenesis and plasticity, whereas NCAM expression levels remain relatively unchanged. When polySia is present on NCAM it acts as a negative regulator of cell interactions associated with a variety of developmental processes that require plasticity, including cell migration, the guidance and targeting of axons and synapse formation.^2, 3^ The loss of polySia-NCAM (polySia attached to NCAM) in NCAM-deficient mice or in wild-type mice treated with a polySia-specific endosialidase has been associated with alterations in a variety of brain functions, including learning and memory behaviors and both long-term potentiation and long-term depression in the hippocampus.^4^ Imbalanced synthesis of polySia and NCAM causes pathological brain development, including reduction of prefrontal cortex (PFC) interneurons, hypoplasia of major brain axon tracts, enlarged ventricles, reduced size of the thalamus and disturbed thalamus–cortex connectivity, which may be linked to the deficits in the cognitive performance observed in these mice.^5, 6, 7^ Furthermore, a synergistic negative consequence of polySia deficiency and cannabis exposure on cognitive performance has been demonstrated,^8^ and in a double-hit animal model of schizophrenia (SZ) prefrontal changes of polySia were detected together with altered excitatory–inhibitory balance.^9^

SZ is a mental disorder that is intimately related to neurodevelopmental and neurodegenerative conditions involving disturbed long-range and synaptic connectivity.^10, 11^ Notably, these abnormalities occur during a very restricted stage of brain development. A number of studies indicate a link between dysregulation of the polySia-NCAM system and SZ.^1, 9, 12, 13^ Altered polySia-NCAM immunoreactivity was observed in schizophrenic hippocampi^14^ and dorsolateral PFC.^9^ This is particularly intriguing because impairment of hippocampal and PFC functions as well as disturbances in their anatomical organization are also involved in the etiology of SZ.^15, 16^ Altered concentrations of NCAM isoforms and soluble NCAM fragments have been frequently observed in the cerebrospinal fluid of SZ patients,^1^ as well as in the hippocampus, PFC and other cortical areas including Brodmann area 46.^17^ Genetic association studies have also identified NCAM and the polySia-synthesizing enzyme *ST8SIA2* as a candidate susceptibility gene for SZ; however, findings are not fully consistent.^9, 12, 13, 18, 19^ Taken together, these findings raise the question of whether polySia-NCAM has a causal role in the development of SZ symptoms.

Because dysregulation of polySia-NCAM accounts for a long-range hypoconnectivity, reduced gray matter (GM) volumes and cognitive deficits in the mouse model,^5, 7^ we hypothesized that polySia-NCAM levels in SZ might be linked to brain structural variations and clinical parameters of cognition and psychopathology. To test this hypothesis, serum levels of polySia-NCAM were assessed in a group of 45 SZ patients and 45 age- and gender-matched healthy controls (HC), and were correlated to macrostructural (volume) and microstructural (diffusion tensor-derived) measures of brain structural integrity as well as to psychopathological and cognitive symptoms in the patient group.

## Materials and methods

### Subjects

As no preliminary data comparing polySia-NCAM between HC and SZ are available, sample numerosity was chosen assuming a Type I error rate *α*=0.05, a medium-high Cohen's effect size of 0.6 and a statistical power of 0.8.^20^ With these assumptions the size of the total sample, HC plus SZ, results to be 90.

For this study we initially included 62 SZ patients consecutively recruited at IRCCS Santa Lucia Foundation of Rome. The diagnosis of SZ was made according to the Diagnostic and Statistical Manual of Mental Disorders IV-Edition, text revised (DSM-IV-TR).^21^ The clinician who had been treating the patients and knew their clinical history, but who was blind to the aims of the study, made the preliminary diagnosis. Then, a senior research psychiatrist confirmed all preliminary diagnoses using the Structured Clinical Interview for DSM-IV-TR-Patient Edition (SCID-I/P).^22^ Out of the original group of patients confidently diagnosed with SZ, four refused to undergo the magnetic resonance imaging exam, seven were excluded for strong movement artifacts in brain images and six were excluded because of the presence of moderate to severe brain vascular lesions (see exclusion criteria below). The remaining 45 SZ patients were age- and gender-matched with 45 HC consecutively recruited from universities, community recreational centers and hospital personnel through local advertisement.

Overall severity of psychiatric symptoms was assessed using the Positive and Negative Syndrome Scale (PANSS).^23^ Age at onset was defined as the age at onset of positive or negative symptoms preceding the first hospitalization, which was investigated in an interview with patients and first-degree relatives.

All patients, but six, were receiving stable oral dosages of one or more atypical antipsychotics such as risperidone, quetiapine and olanzapine. Antipsychotic dosages were converted to equivalents of chlorpromazine.^24^ Extrapyramidal side effects and abnormal involuntary movements were evaluated using the Simpson Angus Scale^25^ and the Abnormal Involuntary Movement Scale.^26^

All HCs were screened for a current or lifetime history of DSM-IV-TR Axis I and II disorders using the SCID-I/NP and SCID-II;^27^ they were also assessed to confirm that no first-degree relative had a history of psychosis.

Inclusion criteria for all participants were as follows: (1) age between 18 and 65 years, (2) at least 8 years of education and (3) suitability for magnetic resonance imaging scanning. Exclusion criteria were as follows: (1) history of alcohol or drug abuse in the 2 years before the assessment, (2) lifetime drug dependence, (3) traumatic head injury with loss of consciousness, (4) past or present major medical illness or neurological disorders, (5) any (for HC) or additional (for SZ) psychiatric disorder or mental retardation, (6) dementia or cognitive deterioration according to DSM-IV-TR criteria and a Mini-Mental State Examination (MMSE)^28^ score lower than 25, consistent with normative data in the Italian population^29^ and (7) any potential brain abnormality and microvascular lesion as apparent on conventional FLAIR scans; in particular, the presence, severity and location of vascular lesions were computed according to the semiautomated method recently published by our group.^30^

Sociodemographic and clinical characteristics of the SZ and HC samples are shown in Table 1.

The study was approved and undertaken in accordance with the guidelines of the Santa Lucia Foundation Ethics Committee. All participants gave their written informed consent to participate after they had received a complete explanation of the study procedures.

### Determination of polySia-NCAM serum levels

Sera were obtained by centrifugation of clotted blood samples, and aliquots were stored at −80 °C. For determination of polySia-NCAM, serum samples were thawed and assayed using a sandwich enzyme-linked immunosorbent assay (modified from Takamatsu *et al.*^31^ and detailed in Supplementary Materials). Each sample was analyzed by at least four independent experiments and values were normalized to the mean value of all measured samples (100%).

### Psychopathological and cognitive assessment

The PANSS^23^ was administered to rate the severity of psychopathological symptoms. The PANSS rates the patient from 1 to 7 on 30 different symptoms based on the interview as well as reports of family members or primary care hospital workers. The symptoms are grouped into three global scales, that is, Positive Symptoms Scale, Negative Symptoms Scale and General Psychopathology Scale, and five subscales, that is, Paranoid-Belligerence, Anergia, Depression, Activation and Thought Disturbance. As 1, rather than 0, is given as the lowest score for each item, a patient cannot score lower than 30 for the total PANSS score. PANSS ratings were obtained on all information available pertaining to the last week of the assessment. Aggressive behavior was assessed through the administration of the Modified Overt Aggression Scale.^32^

With regard to the neuropsychological assessment, the MMSE was administered to obtain a global index of cognitive deterioration. Several tests were selected to provide information about the functioning of different cognitive domains such as: verbal memory (Mental Deterioration Battery (MDB) Rey's 15-word Immediate Recall and Delayed Recall); short-term visual memory (MDB Immediate Visual Memory); logical reasoning (MDB Raven's Progressive Matrices' 47); simple constructional praxis (MDB Copying Drawings and Copying Drawings with Landmarks); language (MDB Phonological Verbal Fluency and Category Fluency);^33^ executive functions (Modified Wisconsin Card Sorting test);^33^ and divided attention and attentional control (Double Barrage test).^34^ We also administered the Rey–Osterrieth Complex Figure Test immediate copy^35^ to evaluate visuo-constructive abilities.

### Image acquisition and processing

All the 90 participants underwent the same imaging protocol, which included three-dimensional T1-weighted, DTI, T2-weighted and FLAIR sequences, using a 3T Allegra MR imager (Siemens, Erlangen, Germany) with a standard quadrature head coil.

T1 and DTI images were processed in order to obtain GM and white matter (WM) volumetric maps as well as fractional anisotropy and mean diffusivity maps. Details about the image-processing methodology can be found in the Supplementary Materials.

### Statistical analyses—clinical and polySia-NCAM

Differences between diagnostic groups on sociodemographic, clinical and biological variables were assessed by means of unpaired *t*-tests. Relationships among continuous variables were analyzed by Pearson's product moment correlations (Fisher's *r* to *z* transformations). The statistical threshold was set at *P*<0.05.

### Statistical analyses—neuroimaging

In order to avoid possible edge effects between different tissue types, the voxel-based morphometry analyses of GM and WM volumes were carried out by excluding all voxels that had a less than 20% probability of belonging to the relative tissue (absolute threshold masking). Further, analyses of mean diffusivity maps were restricted to cortical and deep GM structures by means of an inclusive mask obtained by averaging subjects' GM segments and excluding all voxels with a less than 30% probability of belonging to GM. Finally, statistical analyses on fractional anisotropy maps were restricted to voxels in the WM skeleton.

Although a parametric approach within the SPM8 framework was used for volumetric data, DTI analyses were carried out with a nonparametric permutation-based methodology (that is, the randomize tool, implemented in FSL).

The differential relationship between polySia-NCAM and neuroimaging parameters in the two HC and SZ groups was assessed as follows: subjects' polySia-NCAM levels were entered in four analysis of covariance designs (one for each parameter) as continuous variables, thus modeling the main effect of group (treated as independent variable) and its interaction with polySia-NCAM levels on each neuroimaging parameter (GM and WM volume, GM mean diffusivity and WM tracts fractional anisotropy). Positive (polySia-NCAM_HC_–polySia-NCAM_SZ_) and negative (polySia-NCAM_SZ_–polySia-NCAM_HC_) interaction contrasts were defined to assess the differential relationship between polySia-NCAM levels and neuroimaging parameters in the two HC and SZ groups. MMSE scores were entered in the model as nuisance variable.

The statistical threshold was set at *P*<0.05 using a family-wise error correction that accounted for multiple comparisons and a cluster-size threshold of 30 contiguous voxels. The mean volumetric values of the brain areas, where significant effects were found, were extracted for each subject using an in-house shell script. These values were subsequently used to create scatterplots showing the direction of the relationship.

## Results

As shown in Table 1, the two groups were perfectly matched for gender distribution (31 male subjects in both groups) and did not differ for age; HC had more years of formal education and higher MMSE scores.

SZ showed higher levels of polySia-NCAM. This did not seem to be driven by treatment because no correlation was found between polySia-NCAM levels and antipsychotic drug dosages in equivalents of chlorpromazine (*r*=0.024; *P*=0.822). We also computed the relationship between polySia-NCAM levels and severity of positive and negative symptoms as well as cognitive performance of the SZ sample. We found a significant positive correlation among polySia-NCAM levels, the PANSS-negative scale score (*r*=0.317; *P*=0.033) and the PANSS-blunted affect score (*r*=0.304; *P*=0.041) as well as a significant negative correlation between polySia-NCAM and delayed recall of the Rey–Osterrieth Complex Figure Test score (*r*=−0.321; *P*=0.032). No significant correlations were found between polySia-NCAM levels and positive symptoms as well as Modified Overt Aggression Scale scores.

**Figure 1:**
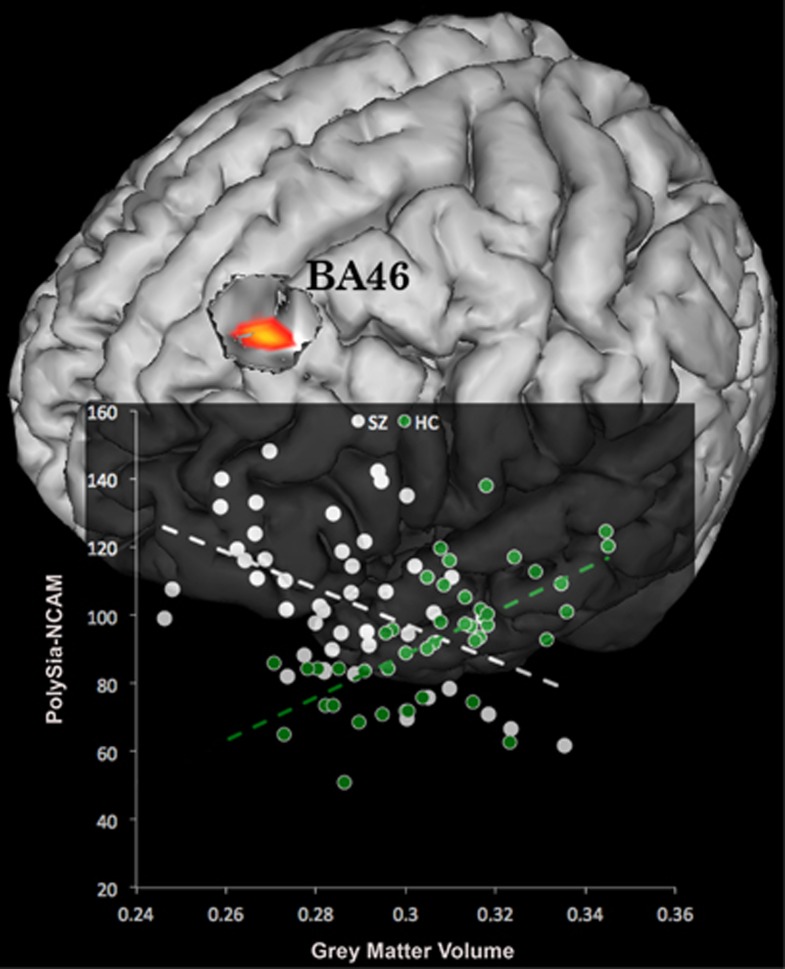
Relationship between neural cell adhesion molecule (NCAM) serum levels and gray matter volume in healthy control (HC) and schizophrenia (SZ). The statistical result (red-to-yellow map) is superimposed over a three-dimensional rendering of the Montreal Neurological Institute standard brain. A scatterplot shows the differential relationship between NCAM and volume in the two study groups.

With respect to the neuroimaging analyses, we found a statistically significant (*P*<0.05, family-wise error-corrected) interaction between the *diagnostic group* variable and polySia-NCAM on GM volume in a prefrontal cortical area. Namely, in a cluster located in the left PFC (Montreal Neurological Institute coordinates: *x*=−16; *y*=36; *z*=27; Brodmann area 46; *Z*-score=4.68; extent: 218 voxels), where SZ patients showed a negative relationship between GM volume and polySia-NCAM levels, whereas HC showed a positive association (see Figure 1a).

Regarding WM volumetric analyses, as well as WM fractional anisotropy and GM mean diffusivity, no results survived the *P*<0.05, family-wise error-corrected, threshold.

## Discussion

The aim of the present study was to investigate whether polySia-NCAM serum levels in SZ patients are different from those of HC and whether they are associated with brain macro- and microstructural variations and psychopathological-cognitive symptomatology. To the best of our knowledge, this is the first study investigating this issue and, therefore, our work could provide an additional piece for the complex puzzle of research on the biological mechanisms implied in SZ pathogenesis. SZ is a psychiatric disorder with a complex pathophysiology that is influenced by multiple factors. It is believed that SZ is highly related to both neurodevelopmental and neurodegenerative processes involving disconnectivity and disorder of synapses.^36^ Thus, several factors are intricately involved in the pathophysiology of SZ and make it difficult to decipher the underlying mechanisms. Nevertheless, the unprecedented correlations of polySia-NCAM serum levels with brain structural variation and, at the same time, with negative symptoms of schizophrenic patients (as demonstrated by the current study) strongly suggest that dysregulation of polySia-NCAM is an important molecular mechanism in the pathophysiology of the disorder.

We found that polySia-NCAM levels were more elevated in SZ patients when compared with HC and that they were differently associated with GM volume in the two groups. Namely, increased polySia-NCAM levels were linked to greater volumes in the HC sample and were associated with reduced volumes in SZ patients. This reverse relationship of polySia-NCAM serum levels and brain integrity was found in a cortical area (that is, the left PFC (Brodmann area 46)), whose early alteration is one of the most replicated findings in SZ. In particular, as recently reviewed by Thermenos *et al.*,^37^ there is significant evidence of reduced PFC volume in high-risk and early SZ patients that becomes more significant over time and is associated with higher symptom severity. Post-mortem studies are consistent with the likelihood of primary structural abnormalities, possibly accounting for alteration of the PFC in SZ. Indeed, findings of increased cell packing density without a change in neuronal number suggest that PFC volume reduction may be linked to a decreased amount of neuropil in SZ.^38, 39, 40, 41^ This is mirrored by functional alteration in the PFC observed across a variety of cognitive tasks (for example, working memory, semantic encoding and language tasks), symptoms and variance in SZ risk genes. Such PFC abnormalities seem to be associated with developmental processes that could be linked to polySia-NCAM alterations. In fact, it has been suggested that abnormal early neurodevelopment (whether linked to genetic or environmental factors or both) may impair the process of normal maturational changes that occurs in adolescence (for example, synaptic remodeling,^42^ WM development^43^ and sensitivity to effects of stress and sex hormones^44^). The final development of the PFC in late adolescence and young adulthood^45^ may be particularly at risk, with adverse implications for both PFC structure and function including maturation of activity, connectivity and network topology.^46, 47^ To date, a range of evidence suggests that macroscopic changes in GM volume over time are not because of the degeneration of neurons, but may reflect (at least in part) the end point of a longstanding disturbance in the plastic rearrangement of the neural connectivity architecture, especially at the level of synapses and prefrontal cortical connections.^48^ In this view, the altered expression of polySia-NCAM may be linked to neurodevelopment and neuroplasticity processes, ultimately leading to changes in the volume of brain areas such as the PFC, where altered concentrations of polySia and NCAM isoforms have been previously found in SZ patients.^9, 17^ In summary, because neurodevelopmental mechanisms affect brain structure before the onset of psychosis and continue throughout the progression of the disease, the polySia-NCAM changes may not be involved only in neurodevelopmental mechanisms, as evident from animal models.^49^ Ongoing dysregulation of polySia-NCAM, as evidenced by the correlation of polySia-NCAM serum levels with altered brain structure in SZ but not HC, may also be relevant for the mechanisms that cause the disease-specific progression of brain structural changes.

Further, coherently with imaging data we found that polySia-NCAM levels were associated with increased negative symptoms and blunted affect (that is, diminished emotional responsiveness, as characterized by a reduction in facial expression, modulation of feelings and communicative gestures) as well as impaired long-term visual–spatial memory. These findings are in line with several previous reports in both animal and human studies. As stated, polySia-NCAM is involved in several neurodevelopmental processes and has been suggested to have a role in the negative and cognitive symptoms of SZ, which are known to present early in the course of the illness.^49^ For instance, NCAM−/− mice display a 10% reduction in the overall brain weight and a 36% decline in the size of the olfactory bulb as well as impaired spatial learning.^50^ Using NCAM-180 knockout mice, Wood *et al.*^51^ demonstrated a marked increase in both the left and right anterior horns of the lateral ventricles and reduced prepulse inhibition, a behavioral model of SZ. Furthermore, reduced polysialylation causes pathological brain development comprising reductions of parvalbumin-positive interneurons in the PFC^6^ as well as enlarged lateral ventricles, smaller thalamus and reduced thalamocortical connectivity together with impaired working memory and prepulse inhibition.^7^

Conversely to negative symptoms, in our sample polySia-NCAM serum levels did not result to be linked to positive symptomatology or aggressive behavior, whereas previous animal studies using both deficient and postnatally treated mice showed a link between polySia-NACM defect and increased aggressive behavior.^52, 53^ A possible rationale accounting for this discrepancy relies in the fact that in these previous studies stress was usually experimentally manipulated, and resulted to significantly interact with polySia-NCAM decreased expression in modulating aggressive and emotional behavior.

The increased polySia-NCAM serum levels in SZ patients, such as those detected in the current study, contrast with reports of lower numbers of polySia-positive cells in the hilus region of the hippocampus and reduced polySia immunoreactivity in the PFC of SZ patients.^9, 14^ This discrepancy in results may be explained by previous results. For NCAM, an inverse relationship between levels found in the brain and in the serum was reported in Alzheimer's disease. In particular, serum NCAM levels were increased in patients with high Global Deterioration Scale values,^54^ whereas they were decreased in the frontal and temporal cortex^55^ and significantly fewer NCAM-positive neurons were detected in the frontal cortex.^56^ Therefore, it may well be that the higher polySia-NCAM serum levels in SZ are linked to altered expression in the brain, possibly reflecting neurodegenerative processes.

Similar to the results of the current study, Lyons *et al.*^57^ found an increase in the NCAM serum levels in SZ patients when compared with normal controls and demonstrated that this difference was more marked in type II SZ (that is, in those patients who showed more negative symptoms). In contrast, a recent study detected significantly lower NCAM serum levels in SZ patients.^58^ Differences in the efficiency of capturing and/or detecting different NCAM species could be responsible for these seemingly divergent results. Furthermore, it is well known that recognition of NCAM is strongly reduced in the presence of polySia.^59^ Hence, the elevated polySia-NCAM levels of SZ patients found in the current study could cause reduced NCAM detection, as observed by Zhang *et al.*^58^ In addition to these reciprocal findings for NCAM and polySia-NCAM, previous report indicated that the serum levels are inversely related to those found in the brains of SZ patients. Also, reduced polySia immunoreactivity in the PFC and hippocampus, as discussed above, contrasts with an increase in NCAM fragments in the same brain areas.^60^ Furthermore, in a study on a large sample of SZ patients (*n*=641) Sullivan *et al.*^61^ found an association between five contiguous single-nucleotide polymorphisms (rs1943620, rs1836796, rs1821693, rs686050 and rs584427, associated with gene *NCAM1*) and cognitive functioning. Thus, the present data seem to support the view that polySia-NCAM abnormalities in SZ are strongly linked to volume reduction in PFC and, coherently, with negative symptoms and cognitive disorders.

SZ is a complex disease in which both biological and environmental factors contribute to the pathogenesis. It is therefore likely that the mechanisms through which altered polySia-NCAM serum levels are connected to the disorder are also complex. Nevertheless, altered serum levels, such as those detected in the current study, may be indicative of disturbed polySia-NCAM homeostasis, which, as discussed above, has the potential to profoundly affect brain development and connectivity. The clear correlations of polySia-NCAM serum levels with altered brain structural measures underscore this link. In parallel, the intriguing result of a positive correlation between polySia-NCAM and prefrontal volume in the HC sample claims in favor of the proven relationship between polySia-NCAM expression and adult neuroplasticity. This is suggested by the remarkable amounts within the main adult neurogenic areas such as the subventricular zone, the olfactory bulb and the dentate gyrus, as well as by the reciprocal relationship between polySia-NCAM and learning and memory.^62^

A final point merits further discussion, namely the absence of correlation between polySia-NCAM levels and antipsychotic drug dosages. This is in partial disagreement with previous findings showing a significant and selective relationship between polySia-NCAM and antipsychotic treatment. In particular, Frasca *et al.*^63^ performed a 2-week treatment with the first-generation antipsychotic haloperidol and the second-generation antipsychotic olanzapine and analyzed the expression of polySia-NCAM in the rat hippocampus and PFC via immunohistochemistry. Results showed a regional- and drug-selective increase in the polySia-NCAM expression in PFC of olanzapine-treated rats with no effects in the hippocampus; conversely, haloperidol did not produce a change in either brain region. However, these results are hardly comparable to those obtained in our study for several reasons. First, rats used in the work of Frasca *et al.* were not animal model of SZ and, as such, results on polySia-NCAM expression can only be linked to the influence of treatment and not to the possible role of the disease *per se*. Second, our SZ sample was composed of patients with long duration of illness and thus were chronically treated, whereas rats received a 2-week treatment. Therefore, it could be the case that the results of Frasca *et al.* highlight the early influence of antipsychotic treatment on polySia-NCAM expression thus overlooking potential long-term effects. Future studies should be specifically designed to unravel this key issue.

In conclusion, as demonstrated by the present study, exploring the mechanisms that establish the link between altered polySia-NCAM levels and behavioral and neuroimaging data should provide further insight into SZ diagnosis, prognosis and treatment.

## Figures and Tables

**Table 1:** Sociodemographic and clinical characteristics of HC and SZ patients

*Characteristics*	*HC (*n=*45)*	*SZ (*n=*45)*	t	P
Age (years±s.d.)	39.4±10.9	37.6±9.5	0.848	0.39
Males *n* (%)	31 (68.8)	31 (68.8)	0.00	>0.999
Educational level (years±s.d.)	16.4±3.2	12.5±3	5.9	**<0.001**
PolySia-NCAM (rel. levels, mean±s.d.)	92.5±18.5	104.6±21.7	2.858	**0.005**
MMSE (mean±s.d.)	29.4±0.8	27.6±2.8	4.167	**<0.001**
Chlorpromazine equivalents (mg per day)	—	237.5±581.3	—	—
PANSS positive	—	21.7±6.4	—	—
PANSS negative	—	18.7±7.3	—	—
PANSS general psychopathology	—	43.8±11	—	—

Abbreviations: HC, healthy controls; MMSE, Mini-Mental State Examination; PANSS, Positive and Negative Syndrome Scale; SZ, schizophrenia. Bold values indicate statistically significant differences.
