# Supplementary material for: Brain structure, cognition and negative symptoms in schizophrenia are associated with serum levels of polysialic acid-modified NCAM
Source: Transl Psychiatry. 2015 Oct 13;5(10):e658–. doi: 10.1038/tp.2015.156 (PMC4930132; doi:10.1038/tp.2015.156)
Supplement: Supplementary Information [file tp2015156x1.docx]

**Determination of polySia-NCAM serum levels**

PolySia-NCAM serum levels were determined by sandwich ELISA using the polySia-specific monoclonal antibody (mAb) 735 ^1^ for capture and horseradish peroxidase-conjugated anti-human NCAM antibody SCLC1 for detection (kindly provided by the Research Laboratories of Dade Behring Marburg GmbH, Marburg, Germany). 96 well half area microplates (Greiner Bio-one, Frickenhausen, Germany) were coated with 5 µg/ml mAb 735 in a volume of 25 µl per well for 1h at room temperature (RT) and blocked with 0.1 M phosphate buffer pH 7.25/0.1% Tween 20 (PBT) containing 1% bovine serum albumine (BSA; 100 µl per well, overnight at 4°C). After washing, 10 µl per serum sample were incubated for 1h at RT followed by washing and incubation with 100ng/ml horseradish peroxidase-conjugated SCLC1 antibody in BSA/PBS (10µl per well) for 1h at RT. After further washing, 25 µl of citrate buffer, pH 4.9 containing 0.01% H_2_O_2_ and 1 mg/ml 3,3’,5,5’-tetramethylbenzidin (TMB; Sigma, Deisendorf, Germany) were added per well. After 20 min, the reaction was stopped by the addition of 10 µl 1M H_2_SO_4_ and absorbances were determined at 450 nm against a reference wavelength (550 nm). A standard curve prepared with polySia-NCAM (kindly provided by the Research Laboratories of Dade Behring Marburg GmbH) verified a linear relationship between amounts of polySia-NCAM and the absorbance at 405 nm. Controls without serum as well as samples treated with endosialidase (10 µg/ml, 1h on ice) in order to specifically degrade polySia ^2^ showed absorption levels <1% of the values for untreated serum samples.

**Image Acquisition and Processing**

All 90 participants underwent the same imaging protocol, which included 3D T1-weighted, DTI, T2-weighted and FLAIR sequences, using a 3T Allegra MR imager (Siemens, Erlangen, Germany) with a standard quadrature head coil.

T1 and DTI images were processed in order to obtain GM and white matter (WM) volumetric maps as well as fractional anisotropy (FA) and mean diffusivity (MD) maps. Detailed image processing methodology can be found in the Supplementary Materials.

Whole-brain T1-weighted images were obtained in the sagittal plane using a modified driven equilibrium Fourier transform sequence (TE/TR = 2.4/7.92 ms, flip angle 15°, voxel size 1×1×1 mm3) (MDEFT). Diffusion-weighted volumes were acquired using spin-echo EPI (TE/TR = 89/8500 ms, bandwidth = 2126 Hz/vx; matrix size 128×128; 80 axial slices, voxel size 1.8×1.8×1.8 mm3) with 30 isotropically distributed orientations for the diffusion sensitising gradients at a b-value of 1000 s/mm2 and 2 no diffusion weighted images (b0). Scanning was repeated three times to increase the signal-to-noise ratio. T2 and FLAIR sequences were acquired to screen for brain pathology.

T1-weighted and DTI images were processed separately in order to obtain indices of brain macro (volume) and micro (diffusivity and anisotropy) structural integrity.

T1-weighted images were processed and examined by using the SPM8 software (Wellcome Department of Imaging Neuroscience Group, London, UK; http://www.fil.ion.ucl.ac.uk/spm), specifically the VBM8 toolbox (available at http://dbm.neuro.unijena.de/vbm.html), running in Matlab 2007b (MathWorks, Natick, MA, USA). The toolbox extends the unified segmentation model ^3^ consisting of MRI field intensity inhomogeneity correction, spatial normalization and tissue segmentation at several pre-processing steps to further improve the quality of data pre-processing. Initially, to increase the signal-to-noise ratio in the data, an optimized block wise nonlocal-means filter was applied to the MRI scans using the Rician noise adaption ^4^. Then, an adaptive maximum a posteriori segmentation approach extended by partial volume estimation was employed to separate the MRI scans into GM, WM and Cerebro-Spinal Fluid (CSF). The segmentation step was finished by applying a spatial constraint to the segmented tissue probability maps based on a hidden Markow Random Field model to remove isolated voxels which were unlikely to be a member of a certain tissue class and to close holes in clusters of connected voxels of a certain class, resulting in a higher signal-to-noise ratio of the final tissue probability maps. Then, the iterative highdimensional normalization approach provided by the Diffeomorphic Anatomical Registration Through Exponentiated Lie Algebra ^5^ (DARTEL) toolbox was applied to the segmented tissue maps in order to register them to the stereotactic space of the Montreal Neurological Institute (MNI). The tissue deformations were used to modulate participants' GM and WM tissue maps. Voxel values of the resulting normalized and modulated GM and WM segments indicated the probability (between 0 and 1) that a specific voxel belonged to the relative tissue. The modulated and normalized GM and WM segments were written with VBM8 standard isotropic voxel resolution of 1.5 mm3 and smoothed with a 6 mm FWHM Gaussian kernel, thus obeying the ‘rule of thumb’ that the FWHM should be at least twice the voxel dimension in order to ensure a Gaussian distribution of the residuals of the General Linear Model ^6^. The segmented, normalized, modulated and smoothed GM and WM images were used for analyses.

DTI images were processed using FSL 4.1 software (www.fmrib.ox.ac.uk/fsl/). Images were corrected for the distortion induced by eddy currents and head motions, by applying a 3D full affine alignment of each image to the mean b0 image.

After distortion corrections, DTI data were averaged and concatenated into 31 (1 b0+30 b1000) volumes. A diffusion tensor model was fit at each voxel, generating fractional anisotropy (FA) and mean diffusivity (MD) maps.

We used Tract-Based Spatial Statistic (TBSS) ^7^ version 1.2, part of FSL for the post processing and analysis of FA maps in WM. First, FA images were created by fitting a tensor model to the raw diffusion data using FDT, and then brain-extracted using BET ^8^. All subjects' FA data were then aligned into the standard MNI space using the nonlinear registration tool FNIRT ^9,10^ which uses a b-spline representation of the registration warp field ^11^. Next, the mean FA image was created and thinned to create a mean FA skeleton which represents the centres of all tracts common to the group. Each subject's aligned FA data was then projected onto this skeleton and the resulting data fed into voxelwise cross-subject statistics ^12^.

MD maps were registered onto the standard space by applying the registration parameters used for FA alignment.

To obtain fine anatomical-connectivity localization of statistical results, two different brain atlases were used: i) the Automated Anatomical Labeling (AAL) ^13^ which includes all main gyri and sulci of the cerebral cortex and the subcortical and deep gray matter structures for a total of 90 anatomical volumes of interest and ii) the ICBM-DTI-81 white-matter labels atlas ^14^, which includes 50 white matter tract labels created by hand segmentation of a standard-space average of diffusion MRI tensor maps from 81 subjects.

**References**

1 Frosch M, Görgen I, Boulnois GJ, Timmis KN, Bitter-Suermann D. NZB mouse system for production of monoclonal antibodies to weak bacterial antigens: isolation of an IgG antibody to the polysaccharide capsules of Escherichia coli K1 and group B meningococci. *Proc Natl Acad Sci U S A* 1985; **82**: 1194–8.

2 Stummeyer K, Dickmanns A, Mühlenhoff M, Gerardy-Schahn R, Ficner R. Crystal structure of the polysialic acid-degrading endosialidase of bacteriophage K1F. *Nat Struct Mol Biol* 2005; **12**: 90–96.

3 Ashburner J, Friston KJ. Unified segmentation. *Neuroimage* 2005; **26**: 839–51.

4 Wiest-Daesslé N, Prima S, Coupé P, Morrissey SP, Barillot C. Rician noise removal by non-Local Means filtering for low signal-to-noise ratio MRI: applications to DT-MRI. *Med Image Comput Comput Assist Interv* 2008; **11**: 171–179.

5 Ashburner J. A fast diffeomorphic image registration algorithm. *Neuroimage* 2007; **38**: 95–113.

6 Moraschi M, Hagberg GE, Di Paola M, Spalletta G, Maraviglia B, Giove F. Smoothing that does not blur: effects of the anisotropic approach for evaluating diffusion tensor imaging data in the clinic. *J Magn Reson Imaging* 2010; **31**: 690–697.

7 Smith S, Jenkinson M, Johansen-Berg H. Tract-based spatial statistics: voxelwise analysis of multi-subject diffusion data. *Neuroimage* 2006; **31**: 1487–1505.

8 Smith SM. Fast robust automated brain extraction. *Hum Brain Mapp* 2002; **17**: 143–55.

9 Andersson JLR, Jenkinson M, Smith S. Non-linear registration aka Spatial normalisation. FMRIB Technial Report TR07JA2. Oxford, United Kingdom, 2007.

10 Andersson JLR, Jenkinson M, Smith S. Non-linear optimisation. FMRIB Technial Report TR07JA1. 2007.

11 Rueckert D, Sonoda LI, Hayes C, Hill DL, Leach MO, Hawkes DJ. Nonrigid registration using free-form deformations: application to breast MR images. *IEEE Trans Med Imaging* 1999; **18**: 712–721.

12 Nichols TE, Holmes AP. Nonparametric permutation tests for functional neuroimaging: a primer with examples. *Hum Brain Mapp* 2002; **15**: 1–25.

13 Tzourio-Mazoyer N, Landeau B, Papathanassiou D, Crivello F, Etard O, Delcroix N *et al.* Automated anatomical labeling of activations in SPM using a macroscopic anatomical parcellation of the MNI MRI single-subject brain. *Neuroimage* 2002; **15**: 273–89.

14 Mori S, Oishi K, Jiang H, Jiang L, Li X, Akhter K *et al.* Stereotaxic white matter atlas based on diffusion tensor imaging in an ICBM template. *Neuroimage* 2008; **40**: 570–82.
